# Colocalization of connexin 36 and corticotropin-releasing hormone in the mouse brain

**DOI:** 10.1186/1471-2202-10-41

**Published:** 2009-04-30

**Authors:** Lars Westberg, Evelyn Sawa, Alice Y Wang, Lisa A Gunaydin, Ana C Ribeiro, Donald W Pfaff

**Affiliations:** 1 Laboratory of Neurobiology and Behavior, The Rockefeller University, 1230 York Avenue, NY10021, New York, USA

## Abstract

**Background:**

Gap junction proteins, connexins, are expressed in most endocrine and exocrine glands in the body and are at least in some glands crucial for the hormonal secretion. To what extent connexins are expressed in neurons releasing hormones or neuropeptides from or within the central nervous system is, however, unknown. Previous studies provide indirect evidence for gap junction coupling between subsets of neuropeptide-containing neurons in the paraventricular nucleus (PVN) of the hypothalamus. Here we employ double labeling and retrograde tracing methods to investigate to what extent neuroendocrine and neuropeptide-containing neurons of the hypothalamus and brainstem express the neuronal gap junction protein connexin 36.

**Results:**

Western blot analysis showed that connexin 36 is expressed in the PVN. In bacterial artificial chromosome transgenic mice, which specifically express the reporter gene Enhanced Green Fluorescent Protein (EGFP) under the control of the connexin 36 gene promoter, EGFP expression was detected in magnocellular (neuroendocrine) and in parvocellular neurons of the PVN. Although no EGFP/connexin36 expression was seen in neurons containing oxytocin or vasopressin, EGFP/connexin36 was found in subsets of PVN neurons containing corticotropin-releasing hormone (CRH), and in somatostatin neurons located along the third ventricle. Moreover, CRH neurons in brainstem areas, including the lateral parabrachial nucleus, also expressed EGFP/connexin 36.

**Conclusion:**

Our data indicate that connexin 36 is expressed in subsets of neuroendocrine and CRH neurons in specific nuclei of the hypothalamus and brainstem.

## Background

Emerging evidence supports a role for gap junctions, intercellular channels that permit a direct exchange of small molecules between adjacent cells, in secretion of hormones [1]. Gap junctions are composed of protein subunits called connexins, which are encoded by a gene family with more than 20 members in mammals [2] and are expressed in a majority of organs, including most of the endocrine and exocrine glands in the body [1]. At least 10 connexins with differing cell specificities are expressed in mammalian nervous systems; connexin 36 [3,4], connexin 45 [5] and connexin 30.2 [6] are considered to be preferentially expressed by neurons. Interestingly, connexin 36 has also been reported to affect synchronization of pancreatic islets and release of the peptide insulin [7]. To what extent connexin 36 affects hormone and neuropeptide release from and within the central nervous system is, however, unknown.

Neuropeptide-releasing neurons in the hypothalamus and other brain areas are known to participate in coordination of autonomic, endocrine, and behavioral functions maintaining the homeostasis of the organism. The neuropeptides oxytocin, vasopressin, corticotropin-releasing hormone (CRH), and somatostatin, all released from the paraventricular nucleus of the hypothalamus (PVN) into the blood stream and the central nervous system, are all crucial for these functions [8-11].

Temporal patterns of action potentials in PVN neurons are notable; when stimulated the magnocellular oxytocin and vasopressin cells show characteristic changes in electrical activity [12,13]. Pulsatile release of oxytocin, such as that observed during lactation, is achieved by synchronous firing of a fixed population of cells, whereas continuous release of vasopressin involves the asynchronous discharge of a variable number of neurons recruited in proportion to the stimulus intensity. As neuronal gap junctions are essential for synchronous firing in many brain areas [14,15], gap junctions between neuropeptide-containing cells could thus be a possible mechanism to explain the characteristic firing patterns of PVN neurons [16]. Intriguingly, dye-coupling studies and electrophysiological experiments in rats have provided evidence for the presence of gap junctions between neurons in both the PVN and the SON [17-21]. The identity of the proteins that comprise these putative gap junctions has however not been established.

In order to investigate connexin 36 expression within the PVN, we used Western blot analysis. Furthermore, bacterial artificial chromosome (BAC) transgenic mice (EGFP/connexin 36 mice) which specifically express Enhanced Green Fluorescent Protein (EGFP) under the control of the connexin 36 promoter were used to further explore to what extent neuroendocrine as well as neuropeptide-containing cells in hypothalamic and brainstem nuclei contain connexin 36.

## Methods

### Animals

All animal protocols were approved by The Rockefeller University Institutional Animal Care and Use Committee. All animal procedures were performed according to the National Institutes of Health and institutional animal care and use guidelines. Adult female and male Swiss-Webster mice were used for the Western blot experiments. All mice were housed on 12:12-h light/dark cycle (lights on at 11.00 h), and food and water were available *ad libitum*.

The EGFP/connexin 36 mice (a gift from Professor Nathaniel Heintz, The Rockefeller University, New York, NY) was made by homologous recombination of a connexin 36 gene-containing BAC (RP23-222L4) comprising an EGFP insert, according to the strategy previously described [22]. This approach does not interfere with the two functional copies of the connexin 36 gene. As the BAC comprises more than 100 kb of flanking DNA both upstream and downstream of the connexin 36, it contains all regulatory sequences needed for an accurate expression of the connexin 36 gene [23,24].

### Western-blot analysis

Three adult male and three adult female Swiss-Webster mice were decapitated and the following brain regions were dissected according to the procedure previously described [25] and rapidly frozen: PVN, suprachiasmatic nucleus, and the reticular nucleus of the thalamus. Tissues were homogenized in a buffer containing 50 mM Tris-HCl, 10 mM MgCl_2_, 150 mM; NaCl, 1% Triton-X 100, 1 mM sodium orthovanadate, 1 mM phenylmethylsulphonyl fluoride and protease inhibitor cocktail (Roche, Basel, Switzerland). Proteins were separated electrophoretically in 10% polyacrylamide gels and transferred to nitrocellulose membranes (Bio-Rad Laboratories, CA) in standard Tris-glycine transfer buffer. Membranes were blocked for 1 h 30 min at room temperature in Tris Buffered Saline-Tween-20 (TBST) (20 mM Tris-HCl, pH 7.4, 150 mM NaCl with 0.05% Tween-20) containing 5% nonfat milk powder and incubated overnight at 4°C with the goat connexin 36 polyclonal antibody sc-14904 (1:400; sc-14904, lot: F192, Santa Cruz Biotechnology, Santa Cruz, CA) [26] in TBST containing 5% non-fat dry milk. Membranes were then washed four times in TBST during 1 hour, incubated with horseradish peroxidase-conjugated bovine anti-goat IgG (1:10,000; Santa Cruz Biotechnology, Santa Cruz, CA) in TBST for 2 h, and washed three times in TBST. Immunoreactive bands were revealed using enhanced chemiluminescence (Western Lightning; Perkin-Elmer, MA). In order to demonstrate specificity of connexin 36 immunoreactivity, primary antibody (4 μg) was pre-incubated with 10× excess of the connexin 36 immunizing peptide (40 μg) (sc-14904P; lot: E0906; Santa Cruz Biotechnology, Santa Cruz, CA) overnight, and the Western blot procedure was repeated on fresh samples. The immunizing peptide sc-14904P has a length of 15–25 amino acids and corresponds to an amino acid stretch of the C-terminus between position 269 and 319 of human connexin 36 (NCBI protein accession number 117688) [26].

### Immunohistochemistry

For immunohistochemistry, animals were anesthetized with sodium pentobarbital and perfused transcardially with 50 mL Phosphate Buffered Saline (PBS) followed by 50–100 mL of 4% paraformaldehyde. Brains were postfixed 2–24 h in the same fixative. After postfixation, brains were stored at 4°C in 30% sucrose for at least 24 h and coronally sectioned at 30 μm on a freezing microtome.

To better visualize cell bodies immunoreactive for CRH and somatostatin in the brain, four EGFP/connexin 36 mice were treated with colchicine (50 pg; right lateral ventricle; 48-hr survival) before perfusion.

In order to label neuroendocrine cells in the brain, 3 male and 3 female EGFP/connexin 36 mice were injected (i.p.) with 30 μl of 4% Fluorogold in 0.9% saline and sacrified 5 days later. As the Fluorogold does not cross the blood-brain barrier, this procedure only labels cells in the brain that project to areas that come into contact with fenestrated capillaries [27]. The brain was perfused and processed using the same protocols as for the other EGFP/connexin 36 mice.

Free floating sections were washed in PBS for 3 × 15 min, blocked with 3% normal goat serum with 0.5% Triton X-100 for 1 h at room temperature, and subsequently incubated in primary antibodies at 4°C for 48 hours. The sections were co-incubated with the rabbit anti-GFP (1:2000–5000; A6455, lot 39587A, Molecular Probes, Eugene, OR) and either anti-oxytocin (1:1000; T-5021, lot 050163-3, Peninsula Laboratories, San Carlos, CA), or anti-vasopressin (1:1000; T-5048; lot: 031088-5, Peninsula Laboratories, San Carlos, CA) guinea pig polyclonal primary antibodies [28,29] with subsequent incubation for 1 h at room temperature in Alexa 488 goat anti-rabbit (1:500; Molecular Probes, Eugene, OR) and Rhodamine Red-X donkey anti-guinea pig (1:200; Jackson ImmunoResearch Laboratories, West Grove, PA) secondary antibodies. Alternatively, the chicken anti-GFP polyclonal primary antibody (1:5000, GFP-1020; lot 1223FPO3, Aveslab, Tigard, OR) was incubated together with either anti-CRH (1:1000; T-4037; lot: 970177-1, Peninsula Laboratories, San Carlos, CA), anti-somatostatin-14 (1:1000; T-4103; lot: 010965-8, Peninsula Laboratories, San Carlos, CA), anti-prepro-orexin (1:200; AB3096, lot 23091616, Chemicon, Temecula, CA) [30,31], anti-histidine decarboxylase (1:200; RDI-PRO16045, Research Diagnostics, Concord, MA) [32], anti-tyrosine hydroxylase (1:500; AB152, lot 250407, Chemicon, Temecula, CA) or anti-Fluorogold (1:1000; AB153; lot: 0509010863, Temecula, Chemicon) rabbit polyclonal primary antibodies with subsequent incubation for 1 h at room temperature in Alexa 488 goat anti-chicken (1:500; Molecular Probes, Eugene, OR) and cy3 donkey anti-rabbit (1:200; Jackson ImmunoResearch Laboratories, West Grove, PA) secondary antibodies. After 3 × 15 min washing in PBS the sections were mounted on Superfrost/Plus slides (Fisher Scientific, Pittsburgh, PA), dried in a light-proof dessicator, and coverslipped using ProLong Gold antifade reagent (Molecular Probes, Eugene, OR).

### Tissue analysis

Brain areas, PVN and others, containing neurons expressing CRH, prepro-orexin, and histidine decarboxylase, were defined according to the Paxinos and Watson [33] atlas. Sections from these brain areas were examined using an inverted LSM 510 laser scanning confocal microscope (Zeiss), and the images were captured with Zeiss LSM 510 (version 3.2) software.

## Results

### Expression of connexin 36 protein in the PVN

**Figure 1:**
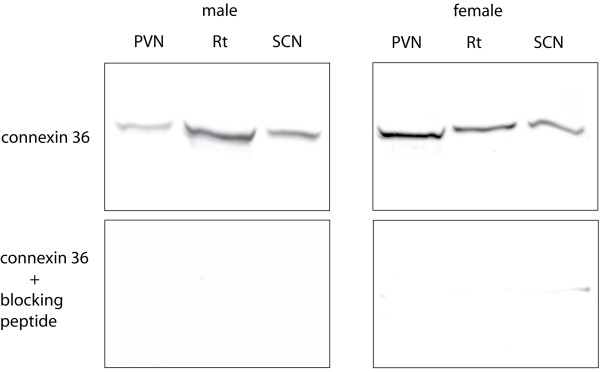
**Top panels: Western blot analysis of connexin 36 protein expression in paraventricular nucleus of hypothalamus (PVN), suprachiasmatic nucleus of hypothalamus (SCN) and the reticular nucleus of the thalamus (Rt) tissue dissected from three male and three female mice**. The molecular weight of the stained band corresponds to 36 kDa. Bottom panels: The immunoreactivity is abolished by pre-incubation with the connexin 36-blocking peptide.

The expression of connexin 36 protein in the PVN was verified by Western blot analysis on tissue dissected and pooled from 3 males and 3 females, and each sex was analyzed separately. Tissue from the reticular nucleus of the thalamus [14] and suprachiasmatic nucleus of the hypothalamus (SCN) [34] was included as a positive control. A single band corresponding to 36 kDa was identified in all three brain regions in males and female mice (Fig. 1B; *upper panels*). The immunoreactivity was abolished by pre-incubation with the blocking peptide (Fig. 1B; *bottom panels*).

### Expression of EGFP from the connexin 36 promoter in the PVN

**Figure 2:**
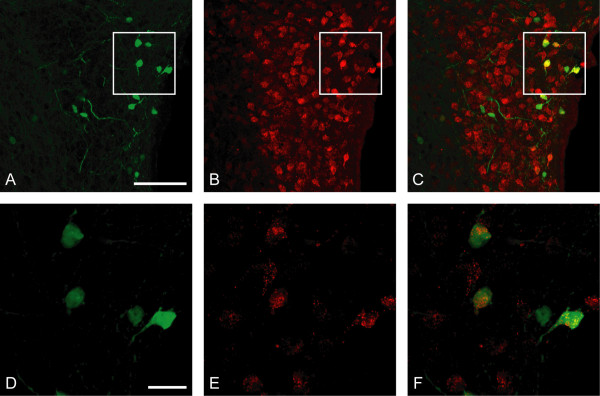
**Expression of EGFP/connexin 36 in a subset of Fluorogold-labeled neuroendocrine cells in PVN**. Confocal photomicrographs show immunofluorescence for EGFP (A, D; *green*), Fluorogold (B, E; *red*), and their colocalization (C; *yellow*) for A, B and (F; *yellow*) for D, E. Photomicrographs D-F show neurons from insets in A-C in higher magnification. The third ventricle is seen to the right in A-C. Scale bars = 100 μm in A-C and 20 μm in D-F.

**Figure 3:**
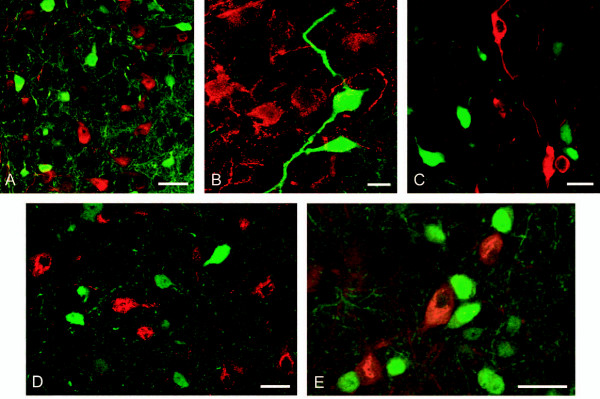
**EGFP/connexin 36 neurons are intermixed with neuropeptide or tyrosine hydroxylase positive cells in hypothalamic nuclei**. EGFP/connexin 36 (*green*) was not colocalized with oxytocin (A; *red*), vasopressin (B; *red*) or tyrosine hydroxylase (C; *red*) neurons in the PVN. Although EGFP/connexin 36 expression (*green*) was seen in the lateral hypothalamus and in the tuberomammillary nucleus, no colocalization between EGFP and prepro-orexin (D; *red*) or between EGFP and histidine decarboxylase (E; *red*) was observed. Scale bars = 20 μm.

**Figure 4:**
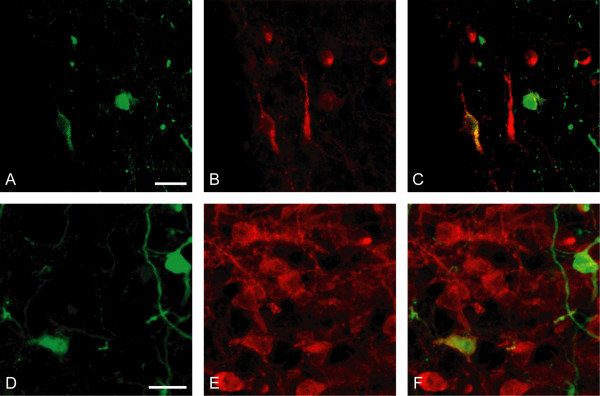
**Some neurons coexpress EGFP/connexin 36 and somatostatin or CRH in the PVN**. (A-C) Confocal photomicrographs show immunofluorescence for EGFP (A; *green*) and somatostatin (B; *red*), and their colocalization (C; *yellow*) in the periventricular zone at the anterior pole of PVN. (D-F) Confocal photomicrographs show immunofluorescence for EGFP (D; *green*) and CRH (E; *red*), and their colocalization (F;*yellow*) in the PVN. The third ventricle is seen to the left in A-C. Scale bars = 20 μm.

Coexpression of connexin 36 with neuropeptides of the PVN was explored using BAC transgenic mice in which the connexin 36 promoter drives EGFP expression. Cells containing EGFP/connexin 36 were scattered through most parts of the PVN. The majority of EGFP-positive cells were small parvocellular neurons (Fig. 2A; 3A), but also some large magnocellular neurons were observed (Fig. 2A; 3B). EGFP-expressing cells were also distributed in the periventricular zone (PeVZ) (Fig. 4A) at most anterior/posterior levels of the hypothalamus.

Following injection of Fluorogold, staining of hypothalamic neuroendocrine cells was observed under UV-light in the PVN, SON, dorsomedial nucleus and arcuate nucleus of hypothalamus in line with previous reports [27]. Fluorogold labeling was detected in a minority of EGFP/connexin 36 expressing neurons of the PVN, and in a majority of EGFP-positive neurons along the third ventricle (Fig. 2).

### Expression of EGFP from the connexin 36 promoter in PVN neurons containing oxytocin, vasopressin, tyrosine hydroxylase, CRH, or somatostatin

No colocalization of EGFP could be seen with oxytocin (Fig. 3A) or vasopressin (Fig. 3B) in the PVN, or with tyrosine hydroxylase (Fig. 3C) in the PeVZ. Previous studies have shown that coupling is relatively infrequent between unstimulated oxytocinergic and vasopressinergic neurons, whereas stimuli such as dehydration [35] and lactation [19,36] increase the level of dye transfer between magnocellular neurons. Therefore, colocalization experiments of EGFP/connexin 36 and oxytocin or vasopressin were also conducted in four EGFP mice dehydrated for 24 hours before sacrifice. However, also in these animals no coexpression between oxytocin or vasopressin and EGFP was detected (data not shown).

As seen in Figure 4 there were some EGFP-positive cells in the PVN/PeVZ that coexpressed CRH or somatostatin. Neurons expressing EGFP and somatostatin were found along the third ventricle mainly in the dorsal parts of PeVZ (Fig. 4A–C), whereas those expressing both EGFP and CRH were localized in medial parts of PVN (Fig. 4D–F).

### Expression of EGFP from the connexin 36 promoter in hypothalamic neurons containing orexin and histamine

Although EGFP/connexin36 and prepro-orexin cells were located in the same areas of the lateral hypothalamus, no colocalization was observed (Fig. 3D). In more posterior regions, EGFP positive cells were seen in tuberomammillary nuclei of the hypothalamus. Although EGFP/connexin 36-positive cells are adjacent to histaminergic cells in the tuberomammillary nuclei, we observed no colocalization with the histamine synthesizing enzyme, histidine decarboxylase (HDC) (Fig. 3E).

### Expression of EGFP/connexin 36 in CRH neurons of extra-hypothalamic nuclei

**Figure 5:**
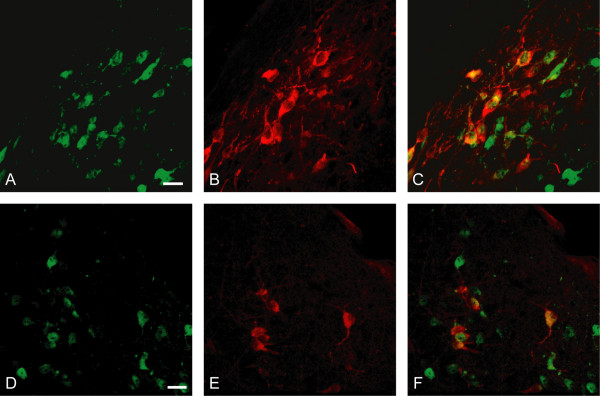
**Expression of EGFP/connexin 36 in CRH neurons of the lateral parabrachial nucleus (A-C) and of the medial vestibular nucleus (D-F)**. Confocal photomicrographs show immunofluorescence for EGFP (A, D; *green*), CRH (B, E; *red*), and their colocalization (C, F; *yellow*). Scale bars = 20 μm.

Since EGFP/connexin 36 colocalized with CRH in the PVN, and it is well known that CRH neurons are distributed throughout the brain, we further explored to what extent EGFP/connexin 36 was expressed in CRH neurons in other brain areas. No double-labeled cells were seen in other hypothalamic nuclei, in the bed nucleus of stria terminalis or in the amygdala. A striking overlap was however seen in the lateral parabrachial nucleus comprising a well-defined group of CRH neurons. A clear majority of CRH neurons in the lateral parabrachial nucleus expressed EGFP (Fig. 5A–C). Colocalization of EGFP and CRH was also seen along the fourth ventricle in the medial vestibular nucleus (Fig. 5D–F) and in the prepositus nucleus (data not shown).

## Discussion

The present study supports previous studies [20,37] showing expression of the neuronal gap junction protein, connexin 36, in the PVN. Our results also provide evidence, for the first time, of colocalization between connexin 36 and CRH in neurons of the PVN and of specific brainstem nuclei.

The connexin 36 protein is a membrane protein shown to be expressed in dendrites rather than in somata of neurons in the brain. Accordingly, previous dye coupling studies indicate a dendritic location of the gap junctions in PVN neurons [16]. Further, available anti-connexin 36 antibodies give rise to a 'punctate' staining pattern, and require delicate experimental conditions in order to work. Similarly, some of the peptides of the current study are mainly located in the axons, which for this study demands specific experimental conditions (i.e. pre-treatment with colchicine). Taken together, these cellular localizations and experimental conditions make it difficult to conduct regular colocalization studies using antibodies against the actual proteins. As the antigenicity of the EGFP protein is maintained during the required experimental conditions we used EGFP/connexin 36 transgenic mice in order to investigate coexpression patterns. These mice have a robust expression of EGFP in the PVN and other brain regions shown to contain connexin 36 protein [37,38].

A large number of studies using dye coupling have provided indirect evidence for gap junction communication between oxytocin and vasopressin containing neurons in the PVN [16,17,20], respectively. Our data using EGFP/connexin 36 transgenic mice suggest that connexin 36 is expressed in neuroendocrine cells of PVN and PeVZ but not in neurons containing oxytocin or vasopressin, neither in unstimulated nor in stimulated animals. This result raises the possibility that other neuronal gap junction proteins may be enabling the proposed communication between magnocellular neurons in the PVN.

As neither electrotonic coupling nor gap junctions have been reported in neurons of the PVN containing CRH, our results showing EGFP/connexin 36 expression in these neurons are intriguing. However, since the number of CRH neurons in the PVN expressing EGFP/connexin 36 is low, the functional relevance of this colocalization is not obvious. Nevertheless, neural net simulations in our laboratory using data modeling suggest that a small subset of coupled neurons can have great implications for the population of neurons they belong to (Weingarten et al, unpublished data). Moreover, as mentioned above, previous studies show that neuronal coupling among PVN neurons is increased by stimulation. To what extent physiological stressors are important for connexin expression and gap junctional coupling in CRH neurons needs to be further explored.

As CRH is expressed in several specific brain nuclei [11] we further investigated if EGFP/connexin 36 was colocalized with CRH in additional brain areas outside the hypothalamus. In contrast to the PVN, where a small subset of CRH neurons were EGFP/connexin 36 positive, a majority of CRH neurons in the lateral parabrachial nucleus also expressed EGFP/connexin 36. Parabrachial neuronal projections are widely distributed throughout the brain [39] and are involved in the regulation of visceral, cardiovascular, respiratory [40] and taste [41] responses. Interestingly, electrical coupling and synchronization of taste-sensitive neurons in parabrachial nucleus has been reported [42]. Furthermore, a considerable overlap between EGFP and CRH was also seen along the fourth ventricle in medial vestibular nucleus and in prepositus nucleus. These brainstem areas are known to comprise CRH neurons [11,43] and have previously been reported to contain connexin 36 [38]. As no overlap between EGFP and CRH was seen in the preoptic area, the bed nucleus of stria terminalis or in the amygdala, a specific role for connexin 36 for CRH neurons in some brain areas, but not in others, is indicated.

Our studies of EGFP-positive neurons in the PeVZ show that most of these cells express somatostatin and project to the bloodstream, that is, they stained for somatostatin and contained the retrograde tracer Fluorogold injected peripherally. To what extent connexin 36 may affect somatostatin release from these neuroendocrine cells, maybe using mechanisms similar to those involved in the release of the peptide hormone insulin from pancreatic islets [7], deserves further study.

The EGFP/connexin 36 expression pattern seen in the lateral hypothalamus and in the tuberomammillary nucleus was in line with previous investigations of connexin 36 mRNA [37,44]. No EGFP expression was however seen in neurons producing orexin or histamine, located in these two areas, respectively. The close proximity of neurons expressing EGFP to neurons producing orexin or histamine is however intriguing and its potential relevance deserves further investigation.

As the EGFP expression of the EGFP/connexin 36 BAC transgenic mice delineates cells expressing connexin 36, but does not provide any information regarding the number or functionality of the connexin proteins of these cells, further studies are necessary to address these issues. In order to confirm our findings indicating colocalization between connexin 36 and CRH in specific brain regions new immunohistochemical protocols, in which these two antigens can be investigated together, must be developed.

## Conclusion

Our results contribute independent data sets indicating that the neuronal gap junction protein connexin 36 is found in the PVN. In EGFP/connexin 36 BAC transgenic mice, subsets of EGFP-positive neurons of the PVN and PeVZ project to the bloodstream, and/or contain somatostatin or CRH. Furthermore, a majority of CRH neurons of the parabrachial nucleus expressed EGFP/connexin 36. The functional implications of these findings need to be addressed in future studies.

## Authors' contributions

LW participated in the design of the study, wrote the first draft of the manuscript, performed immunohistochemistry experiments, did confocal microscopy analysis and bred the transgenic mouse strain. ES conducted immunohistochemistry experiments, did confocal microscopy, and the image analysis. AW conducted immunohistochemistry experiments, as well as the tracing analysis. LG conducted immunohistochemistry experiments. AR did the Western blots and treated mice with colchicine. DP participated in the project's design and coordination and helped to draft and to write the manuscript. All authors read and approved the final manuscript.
